# Poly-Gamma-Glutamic Acid Nanopolymer Effect against Bacterial Biofilms: In Vitro and In Vivo Study

**DOI:** 10.3390/biomedicines12020251

**Published:** 2024-01-23

**Authors:** Eman M. Elsayed, Ahmed A. Farghali, Mohamed I. Zanaty, Medhat Abdel-Fattah, Dalal Hussien M. Alkhalifah, Wael N. Hozzein, Ahmed M. Mahmoud

**Affiliations:** 1 Department of Botany and Microbiology, Faculty of Science, Beni-Suef University, Beni-Suef 62521, Egypt; medhat.mahmoud@science.bsu.edu.eg (M.A.-F.); hozzein29@yahoo.com (W.N.H.); ahmed.mahmoud@science.bsu.edu.eg (A.M.M.); 2 Department of Materials Science and Nanotechnology, Faculty of Postgraduate Studies for Advanced Sciences, Beni-Suef University, Beni-Suef 62521, Egypt; farghali@psas.bsu.edu.eg; 3 Department of Biotechnology and Life Sciences, Faculty of Postgraduate Studies for Advanced Sciences, Beni-Suef University, Beni-Suef 62521, Egypt; zanaty012@psas.bsu.edu.eg; 4 Department of Biology, College of Science, Princess Nourah bint Abdulrahman University, Riyadh 11671, Saudi Arabia; dhalkalifah@pnu.edu.sa

**Keywords:** Ɣ-PGA, nanopolymer, MIC, anti-biofilm, eradication, inhibition, in vivo

## Abstract

In this study, a biodegradable poly-gamma-glutamic-acid nanopolymer (Ɣ-PGA NP) was investigated for its activity against clinical strains of Gram-positive (*Staphylococcus aureus* and *Streptococcus pyogenes*) and Gram-negative (*Klebsiella pneumoniae* and *Escherichia coli*), and reference strains of *S. aureus* ATCC 6538, *S. pyogenes* ATCC 19615 (Gram-positive), and Gram-negative *E. coli* ATCC 25922, and *K. pneumoniae* ATCC 13884 bacterial biofilms. The minimum inhibitory concentration (MIC) effect of Ɣ-PGA NP showed inhibitory effects of 0.2, 0.4, 1.6, and 3.2 μg/mL for *S. pyogenes*, *S. aureus*, *E. coli*, and *K. pneumoniae*, respectively. Also, MIC values were 1.6, 0.8, 0.2, and 0.2 μg/mL for *K. pneumoniae* ATCC 13884, *E. coli* ATCC 25922, *S. aureus* ATCC 6538, and *S. pyogenes* ATCC 19615, respectively. Afterwards, MBEC (minimum biofilm eradication concentration) and MBIC (minimum biofilm inhibitory concentration) were investigated to detect Ɣ-PGA NPs efficiency against the biofilms. MBEC and MBIC increased with increasing Ɣ-PGA NPs concentration or time of exposure. Interestingly, MBIC values were at lower concentrations of Ɣ-PGA NPs than those of MBEC. Moreover, MBEC values showed that *K. pneumoniae* was more resistant to Ɣ-PGA NPs than *E. coli*, *S. aureus*, and *S. pyogenes*, and the same pattern was observed in the reference strains. The most effective results for MBEC were after 48 h, which were 1.6, 0.8, 0.4, and 0.2 µg/mL for *K. pneumoniae*, *E. coli*, *S. aureus*, and *S. pyogenes*, respectively. Moreover, MBIC results were the most impactful after 24 h but some were the same after 48 h. MBIC values after 48 h were 0.2, 0.2, 0.2, and 0.1 μg/mL for *K. pneumoniae*, *E. coli*, *S. aureus*, and *S. pyogenes*, respectively. The most effective results for MBEC were after 24 h, which were 1.6, 0.8, 0.4, and 0.4 µg/mL for *K. pneumoniae* ATCC 13884, *E. coli* ATCC 25922, *S. aureus* ATCC 6538, and *S. pyogenes* ATCC 19615, respectively. Also, MBIC results were the most impactful after an exposure time of 12 h. MBIC values after exposure time of 12 h were 0.4, 0.4, 0.2, and 0.2 μg/mL for *K. pneumoniae* ATCC 13884, *E. coli* ATCC 25922, *S. aureus* ATCC 6538, and *S. pyogenes* ATCC 19615, respectively. Besides that, results were confirmed using confocal laser scanning microscopy (CLSM), which showed a decrease in the number of living cells to 80% and 60% for MBEC and MBIC, respectively, for all the clinical bacterial strains. Moreover, living bacterial cells decreased to 70% at MBEC while decreasing up to 50% at MBIC with all bacterial refence strains. These data justify the CFU quantification. After that, ImageJ software was used to count the attached cells after incubating with the NPs, which proved the variation in live cell count between the manual counting and image analysis methods. Also, a scanning electron microscope (SEM) was used to detect the biofilm architecture after incubation with the Ɣ-PGA NP. In in vivo wound healing experiments, treated wounds of mice showed faster healing (*p* < 0.00001) than both the untreated mice and those that were only wounded, as the bacterial count was eradicated. Briefly, the infected mice were treated faster (*p* < 0.0001) when infected with *S. pyogenes* > *S. aureus* > *E. coli* > *K. pneumoniae*. The same pattern was observed for mice infected with the reference strains. Wound lengths after 2 h showed slightly healing (*p* < 0.001) for the clinical strains, while treatment became more obvious after 72 h > 48 h > 24 h (*p* < 0.0001) as wounds began to heal after 24 h up to 72 h. For reference strains, wound lengths after 2 h started to heal up to 72 h.

## 1. Introduction

The increase in bacterial infections that result in invasive infections has become a dreadful threat to human health [1]. Most bacterial infections of living tissues are associated with biofilms [2,3]. Biofilms are difficult to eradicate because of their three-dimensional structure encapsulated within an extracellular polymeric substance that allows the bacteria to resist antimicrobial agents [4]. The bacterial cells within biofilms are physically protected by a polymeric matrix, and they also change their gene expression to activate resistance mechanisms that protect them from antimicrobial threats [5]. Biofilm infections represent a serious problem for health care systems around the world [6]. Of note, there are no antimicrobial therapies that can target bacterial biofilms, which therefore limits treatment success and increases healthcare costs [7,8]. Therefore, the development of compounds that are capable of eradicating or inhibiting biofilms is desperately required [9].

Most bacterial species could form biofilm [10]. Biofilm formation provides protection to bacterial cells through the extracellular polymeric matrix, causing intrinsic antibacterial resistance. Poor penetration of antimicrobial agents into biofilms is one of the main reasons for the resistance of infectious biofilms to antibacterial treatment [11,12]. Biofilms are highly resistant to antimicrobials and host immune mechanisms, as they could grow under their harboring mechanisms under multiple stress conditions. Hence, it is difficult to treat patients even with high doses of antibiotics [13]. Many studies have focused on bacteriostatic or bactericidal antimicrobial agents to inhibit these resistant microorganisms.

Wound healing is often delayed by bacterial infections that cause tissue inflammation. Once the skin is injured, microorganisms that are normally sequestered on the skin surface obtain access to the underlying tissues. Bacteria in the wounds may form biofilms, especially staphylococci [14].

Wound healing is affected by many factors, including blood clotting, inflammation, and the migration of dermal cells. Wound inflammations due to bacterial infections lead to tissue necrosis and numerous systemic infections [15]. Normal flora on the skin can get access to the underlying tissues once the skin is injured. Bacterial infection can lead to bacterial biofilm formation, which results in a chronic wound state [16]. Biofilms can develop protected environments that shield bacteria from the phagocytosis of neutrophils, which increases their resistance to antibiotics [17]. Biofilm-infected wounds are commonly caused by staphylococci, such as *S. epidermidis* and *S. aureus* [14]. *S. aureus* is one of the most common nosocomial Gram-positive bacteria [18]. *Candidiasis*, *Clostridium*, *Enterococcus*, *Klebsiella*, *E. coli*, and Streptococci are the main causes of infected wounds [19,20,21,22]. The most prevalent Gram-negative biofilm-performing bacterium is *E. coli*. It shows different structural biofilm structures, including simple flat and loose biofilm structures [23]. Therefore, there is an urgent need for effective therapies for the treatment of MRSA biofilm-infected wounds.

Biodegradable polymers have been recommended for various biomedical applications [24,25]. They have shown various applications in several biomedical fields, specifically drug delivery systems, tissue engineering, and wound dressing applications, because of their biocompatible, biodegradable, and nontoxic nature [26,27]. Interestingly, biodegradable polymers include synthetic and natural ones. Synthetic polymers could be used in orthopedic devices due to their good mechanical properties as well as their inability to degrade [28]. In addition to their low degradation rate, natural polymers have good biological and biocompatible properties, so they can be used in medical applications [29].

A wide range of natural polymers, such as chitosan (Ch), hyaluronic acid, chitin, and cellulose-based nanoparticles, have been developed to be compatible with human tissue [30]. These natural polymers have been used for skin regeneration as wound healing dressings in the treatment of various types of wounds [31]. Since slow-healing wounds are a major challenge, antimicrobial agents such as antibiotics and nanoparticles have been uploaded to these polymers for use in medical applications.

Nanoparticles have attracted great attention due to their unique properties. Hence, a lot of nanomedicines and nanostructured materials have been synthesized [32,33,34], including metal oxides, metal nanoparticles, natural nanoparticles, and other types of nanostructures that have shown distinctive antimicrobial properties. In addition, many of these nanoparticles also possess wound-healing properties [35,36].

Previous studies on Ɣ-PGA drug carrier nanoparticles proved their efficiency for microbial inhibition. Cui et al. (2018) proved that nisin-loaded Ɣ-PGA and poly-L-lysine (PLL) nanoparticles inhibited *S. aureus* in pork meat to 1.9 Log CFU/g [37]. Inbaraj et al. (2011) studied the antimicrobial effect of Ɣ-PGA-coated magnetite nanoparticles against *Salmonella enteritidis* SE 01, *E. coli* ATCC 8739, and *S. aureus*. They also proved their non-cytotoxicity against human skin fibroblast cells [38]. Moreover, for wound healing treatments, Wang et al. (2018) proved the efficiency of Ag nanoparticles coated on Ɣ-PGA for in vivo wound healing [39].

Although many nanoparticle types have been used as antimicrobials and some as antibiofilm agents, biofilm infections are still a serious problem. Of note, no previous works studied the Ɣ-PGA NPs effect against biofilms. So, we suggested using Ɣ-PGA NPs, which is a biodegradable polymer previously biosynthesized by the authors [40]. Ɣ-PGA NPs are non-immunogenic homopolymers that prove to be non-cytotoxic NPs.

In this work, we used biodegradable Ɣ-PGA NPs as an antibiofilm agent (biosynthesized by the authors) against Gram-positive and Gram-negative bacteria. This novel antibiofilm NP has a small size (18.63–21.66 nm), which increases its effect against bacterial biofilms. We aimed to apply it as an anti-biofilm agent using practical in vitro methods using different concentrations of the Ɣ-PGA NPs, as well as imaging their anti-biofilm effect. Also, we investigated the in vivo biological response of this NP to skin-infected mice.

## 2. Materials and Methods

### 2.1. Bacterial Strains

Clinically isolated bacteria used in this study were purchased from Faculty of Medicine, Mansoura University, Egypt. They included Gram-positive *S. aureus* and *S. pyogenes* and Gram-negative *K. pneumoniae* and *E. coli*. Additionally, four reference strains were used, namely *S. aureus* ATCC 6538, *S. pyogenes* ATCC 19615 (Gram-positive), *E. coli* ATCC 25922, and *K. pneumoniae* ATCC 13884 (Gram-negative), which were gifted by the National Research Center, Cairo, Egypt.

**Figure 1:**
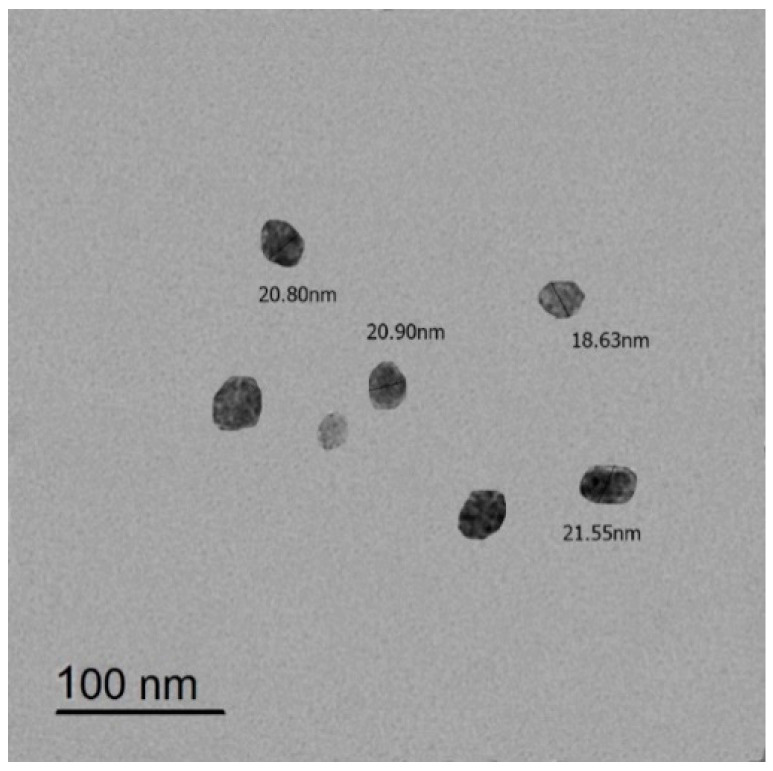
TEM image of Ɣ-PGA NPs.

Ɣ-PGA NP with size of 18.63–21.66 nm (Figure 1) used in the current study was biosynthesized by the authors [40].

### 2.2. Antibacterial Activity of Ɣ-PGA NPs

MIC was assessed separately and according to the NCCLS M7-A8 (2009) standards [41]. Briefly, 10 μL of each bacterial strain (final OD600 = 0.01) was added to 100 μL of varied concentrations of Ɣ-PGA NP (0.1, 0.2, 0.4, 0.8, 1.6, 3.2, 6.4, 12.8, 25.6, and 51.2 µg/mL) in 96-well plates. Three parallel measurements were obtained for each sample. To test the antibacterial activity, all plates were incubated at 37 °C for 24 h, and MIC was defined as the minimum concentration exhibiting no turbidity across the three parallel measurements.

### 2.3. MBIC Technique

Firstly, biofilm formation was assessed qualitatively, as previously described by Christensen et al. [42], with some modifications. Microtiter plates were inoculated with nutrient broth (100 µL) that was inoculated with loops of each bacterial strain from overnight duration culture plates and incubated at 37 °C. The microtiter plates were decanted and washed with PBS. Then, the bacterial biofilms were stained with a crystal violet (CV) stain, SC-ICO diagnostics, Suez, Egypt. Excess stain was discarded, and biofilm formation was considered positive when a visible ring film lined the well and bottom of the wells.

The biofilm inhibition test was achieved as previously described [43]. Briefly, bacterial strains were separately grown overnight at 37 °C in nutrient broth media. Then, 50 μL of each bacterial suspension (OD_570_ = 0.01) was added to the interior wells of a 96-well polystyrene microtiter plate containing 50 μL of different concentrations of the Ɣ-PGA NPs solution (0.1, 0.2, 0.4, 0.8, 1.6, 3.2, 6.4, 12.8, 25.6, and 51.2 µg/mL). Then, the plates were incubated for different periods of time (2, 4, 6, 8, 12, 24, and 48 h) at 37 °C under static conditions. After that, the planktonic cells and the spent medium were discarded, and the adhered biomass was rinsed three times with PBS. Next, plates were washed using distilled water; each well was then stained with 150 μL of 1% CV and kept standing for 15 min at room temperature. Then, the plates were cleaned using sterile water. After that, the residual bound stain was solubilized in 150 μL of 95% ethanol (SPHINX, Cairo, Egypt) for 30 min. Finally, the reduction in biofilm growth was calculated from the absorbance at 570 nm using ELISA (Tecan Group Ltd., Mannedorf, Switzerland). A control well was inoculated with only sterile broth media. Triplicate results were averaged. So, MBIC was detected as the lowest concentration of the NPs that inhibited the development of visible microbial growth [44].

### 2.4. MBEC Technique

To evaluate the effects of antimicrobial agents on the pre-formed biofilms, each bacterial strain was grown separately overnight at 37 °C in nutrient broth media. Then, 20 μL of each bacterial suspension of the resulting culture were transferred to a 96-well microtiter plate. Then, 180 μL of the nutrient broth was added to each well, and the microplates were incubated for 18 h to allow biofilm attachment and growth. After that, the unbound cells were discarded from each well, and the adhered biofilm was rinsed three times with 100 μL PBS. Subsequently, 100 μL of each concentration of Ɣ-PGA NP (0.1, 0.2, 0.4, 0.8, 1.6, 3.2, 6.4, 12.8, 25.6, and 51.2 µg/mL) was added to wells in order. The plates were then incubated for different times (2, 4, 6, 8, 12, 24, and 48 h) at 37 °C under static conditions. After exposure time, the planktonic cells and spent media were discarded, and the remaining biomass was rinsed three times with PBS. Each well was then stained with 150 μL of 1% CV and kept standing for 15 min at room temperature. Then, the plates were cleaned using sterile water. After that, the residual bound stain was solubilized in 150 μL of 95% ethanol for 30 min. Finally, the reduction in biofilm growth was calculated from the absorbance at 570 nm using ELISA (Tecan Group Ltd., Mannedorf, Switzerland). Control well was inoculated with only sterile broth media. Triplicate results were averaged. So, MBEC was detected as the lowest concentration of the NPs that inhibited the development of visible microbial growth [44].

### 2.5. CLSM and SEM Characterization of the Biofilms

Each bacterial strain was cultured overnight and diluted to 1 × 10^6^ CFU/mL using nutrient broth medium. Three groups of bacterial strains were identified. A suspension of 1000 μL of each bacterial strain was inoculated into petri dishes to allow bacterial biofilm adhesion. The first plate group was incubated for 18 h at 37 °C under static conditions. Then, the formed biofilm was washed using PBS, followed by adding 1000 μL of fresh media with the Ɣ-PGA NPs concentration, which eradicated each bacterial biofilm. The second group was incubated with the Ɣ-PGA NPs, which inhibited the bacterial biofilms. After a 24 h incubation, the wells were rinsed three times using PBS to remove planktonic cells. The third group was only bacterial biofilm. Acridine orange and propidium iodide dyes were added for 15 min. at 37 °C for examination by CLSM (LEICA DMi8) at the National Research Center, Cairo, Egypt [8].

For SEM observation, samples were prepared the same as previously prepared CLSM ones. They were fixed using 4% paraformaldehyde overnight without staining, left for drying, gold coated, and imaged using SEM (Gemini Zeiss-Sigma 500 VP, Jena, Germany). Control samples were prepared using untreated bacterial biofilm.

### 2.6. Image Analysis

Live bacterial counts on 2D images of CLSM were quantified using ImageJ processing software (Version 1.46r, National Institutes of Health, Bethesda, MD, USA). This tool enabled us to quantify the bac-light-stained live bacterial cells along some main steps. Images were converted to white scale with a black background. The bright (white) cells were counted after that because this software counts particles on the basis of their relative density (fluorescence) compared with the background via a threshold process. As most bacteria were in aggregates, they were separated by selecting the auto-threshold function to count individual cells. Subsequently, 2D images of control, MBIC, and MBEC ones were counted [45].

### 2.7. Quantification of Adhered Biofilm Cells on Microtiter Plates (CFU)

To measure the live cells adhered within each well of a microtiter plate, biofilms were treated with Ɣ-PGA NPs as mentioned before in Section 2.4 and Section 2.5. for MBIC and MBEC inhibition assays. The nutrient broth medium was removed from all wells after (2, 4, 6, 8, 12, 24, and 48 h). The formed biofilm was washed once with 200 μL of phosphate-buffered saline (PBS). After that, PBS solution (100 μL) was added to wells, and biofilm cells were suspended by vigorous pipetting. Finally, 100 μL each was plated on nutrient agar corresponding to the broths used for biofilm production [8].

### 2.8. Antibiofilm Effect of Ɣ-PGA NP against Mice-Infected Wounds

The experimental protocol was approved by Experimental Animal Ethics Committee of the Faculty of Science, Beni-Suef University, Egypt, for the use and treatment of animals (ethical approval number: 022–325).

Albino mice (about 8–12 months, 30–40 g) were anesthetized with 0.5 mL diethyl ether, and the back skin was injured to induce 0.5 cm diameter skin wounds, which were disinfected with iodine and 70% alcohol for sterile surgery. Cotton pellets containing 1 mL of the bacterial suspension (1 × 10^6^ CFU/mL) were placed over the wound area, covered with plastic wrap, and fixed with a bandage to maintain bacterial growth, thereby biofilm formation. The mice were left for 24 h. Then, wound dressings were prepared by immersing cotton gauze (1.5 × 1.5 cm) into Ɣ-PGA NPs solution to cover the wound sites for different time spans (2, 24, 48, and 72 h). The used Ɣ-PGA NPs concentrations were the same as MBEC concentrations, i.e., 1.6, 0.8, 0.4, and 0.2 µg/mL for *K. pneumoniae*, *E. coli*, *S. aureus*, and *S. pyogenes* infected mice, respectively. After that, sterilized swabs were used to sample the bacteria on the wound surface and spread them on nutrient agar, which was cultured at 37 °C for 24 h to record bacterial density [14,46].

The experiment was designed as follows: there were four mouse groups infected as mentioned above, each with only one pathogenic bacterium: *S. aureus*, *S. pyogenes*, *K. pneumoniae,* or *E. coli*.

Another four groups were infected as mentioned above, then treated using the MBEC concentration of Ɣ-PGA NPs that was detected using the microtiter plate method. Moreover, a group of mice was only wounded to be used as a control. Each group contained six mice [46].

Moreover, the same experimental design was conducted using the four reference strains. Four mouse groups were infected, each with one reference bacterial strain: *S*. *aureus* ATCC 6538, *S. pyogenes* ATCC 19615, *E. coli* ATCC 25922, and *K. pneumoniae* ATCC 13884. Another four groups were infected as mentioned above, then treated using the MBEC concentrations: 1.6, 0.8, 0.4, and 0.4 µg/mL for *K. pneumoniae* ATCC 13884, *E. coli* ATCC 25922, *S. aureus* ATCC 6538, and *S. pyogenes* ATCC 19615, respectively. Also, a group of mice was only wounded to be used as a control. Each group contained six mice.

Injured mice were imaged using a camera, and the wound lengths were measured using a calibrated digital vernier caliper obtained from Metrology lab, Production Technology Dept., Faculty of Technology and Education, Beni-Suef University.

### 2.9. Statistical Analysis

Results were presented as means with the standard deviations (SD) of in vitro results (MIC, MBIC, and MBEC).

For in vivo results, the one-way analysis of variance was used to determine statistical differences between groups (SPSS version 20 software, Chicago, IL, USA). It was used to determine the level of significance, and *p* values less than 0.01 were considered to be significant and highly significant, respectively [47]. Data were tested for normality with Shapiro–Wilk test. Data were not normally distributed, so they were expressed as medians and interquartile ranges (IQR) using Origin software (version 2018).

## 3. Results and Discussion

### 3.1. MIC of Ɣ-PGA NPs

The MIC values of Ɣ-PGA NP against the bacterial strains indicated its antimicrobial activity, as the small size of Ɣ-PGA NPs facilitated easier NP penetration of the cell membrane.

MIC values were reported as arithmetic means; as shown in Table 1, Ɣ-PGA NPs affected *S. pyogenes* at a lower concentration than *S. aureus, E. coli*, and *K. pneumoniae*. As shown in Table 1, MIC values showed the same trend for the reference strains, i.e., Ɣ-PGA NPs affected *S. pyogenes* ATCC 19615 at a lower concentration than *S. aureus* ATCC 6538, *E. coli* ATCC 25922, and *K. pneumoniae* ATCC 13884. Also, the MICs of the reference strains were lower than those of the clinical strains.

Of note, previous works did not study Ɣ-PGA NPs against bacterial strains. Also, some studies focused on loading antibiotics or nanoparticles on polymers. In the same context, Hakkimane et al. (2023) proved that rifampicin (RIF)-loaded poly lactic-co-glycolic acid (PLGA) NPs inhibited the growth of the *Mycobacterium tuberculosis* H37Rv strain at 70% of the MIC of pure RIF (MIC level 1 µg/mL) [48]. So, RIF-encapsulated PLGA NPs were found to be more effective in inhibiting microbial growth than pure RIF.

Also, Durak et al. (2020) found that the MIC values of caffeic acid phenethyl ester and juglone-loaded, multifunctional nano-formulations (polymeric nanoparticles) were 6.25 and 12.5 μg/mL for *S. aureus* and *E. coli*, respectively [49]. Haney et al. (2018) tested the functional gold (CS-Au@MMT) nanocomposites against *E. coli* and *S. aureus,* and the MIC values were 4 μM and 2 μM, respectively [49]. So, they proved that encapsulation of active ingredients into nanoparticles increased their biological activity. These works concluded increasing antimicrobial activity of nanoparticles than other substances.

As discussed by El-Sayed et al. (2017), NPs can enter cells by diffusing through cell membranes. Possible mechanisms include membrane disruption or the potential formation of reactive oxygen species (ROS), protein oxidation, interruption of energy transduction, and the release of toxic constituents [50].

As shown in the present study, the clinical strains are more resistant than the reference ones, which agrees with Mohammadinia et al. (2011), who used disinfecting products against biofilms of the reference strains *Pseudomonas aeruginosa* (ATCC 9027) and *S. aureus* (ATCC 6538) and the clinical isolates of the same strains. They found that the clinical isolates were more resistant than the laboratory ones [51]. This may be due to the widespread use of antibiotics, which caused more bacterial resistance. Shariati et al. (2019) used Ciprofloxacin-Azithromycin nanoparticles on chitosan nanocarriers (Cipro-AZM-Ch) against biofilms of *P. aeruginosa* PAO1 ATCC 27853 and clinical isolates of the same strain. Most of the isolates were extensively more resistant. As a result, they found that Cipro-AZM-Ch was more effective than free Cipro [52]. Fadwa et al. (2021) found that the *P. aeruginosa* ATCC 27853 biofilm was more sensitive to ZnO nanoparticles than clinical isolates [53].

### 3.2. Biofilm Inhibition and Eradication Assays on Microtiter Plates

It was sought to evaluate the capacity of Ɣ-PGA NPs to eradicate, disperse, or inhibit the growth of bacterial biofilms established within the wells of microtiter plates. Antibiofilm activity was evaluated under both eradication (addition after biofilm formation) and inhibition (addition prior to biofilm initiation) conditions against the four selected bacterial biofilms. Bacterial biofilms were mentioned after 12 h for *S. aureus, S. pyogenes*, and *K. pneumoniae* and after 18 h for *E. coli*.

When evaluated under both eradication and inhibitory conditions, results showed that the inhibition of biofilm growth occurred at concentrations higher than the concentrations that inhibited the bacterial growth (MIC) in the previous experiment. As shown in Table 2, the antibiofilm activity of Ɣ-PGA NPs at both MBEC and MBIC increased with the increase in their concentration or the time of exposure. Interestingly, MBEC values indicate the inhibition of biofilm growth occurred at concentrations higher than the concentrations that inhibited bacterial growth (MBIC). That is because of the three-dimensional structure (on the wells) encapsulated within an extracellular polymeric substance after bacterial incubation, which allows the bacteria to resist low concentrations of Ɣ-PGA NPs. At the MBIC test, bacterial strains were incubated with the NPs at the same time, so no bacterial adhesion occurred and lower concentrations could inhibit them.

Moreover, MBEC values showed that *K. pneumoniae* was more resistant to Ɣ-PGA NPs than *E. coli, S. aureus,* and *S. pyogenes.* The most effective results for MBEC were after 48 h, which were 1.6, 0.8, 0.4, and 0.2 µg/mL for *K. pneumoniae*, *E. coli*, *S. aureus*, *and S. pyogenes*, respectively. Moreover, MBIC results were the most impactful after an exposure time of 24 h, but some were the same after 48 h. MBIC values after an exposure time of 48 h were 0.2, 0.2, 0.2, and 0.1 μg/mL for *K. pneumoniae*, *E. coli*, *S. aureus*, and *S. pyogenes*, respectively.

For reference strains, as shown in Table 2, MBEC values were in the same order as those of the clinical strains. So, MBEC values showed that *K. pneumoniae* ATCC 13884 was more resistant to Ɣ-PGA NPs than *E. coli* ATCC 25922, *S. aureus* ATCC 6538, and *S. pyogenes* ATCC 19615. The most effective results for MBEC were after 24 h, which were 1.6, 0.8, 0.4, and 0.4 µg/mL for *K. pneumoniae* ATCC 13884, *E. coli* ATCC 25922, *S. aureus* ATCC 6538, and *S. pyogenes* ATCC 19615, respectively. Also, MBIC results were the most impactful after an exposure time of 12 h. MBIC values after an exposure time of 12 h were 0.4, 0.4, 0.2, and 0.2 μg/mL for *K. pneumoniae* ATCC 13884, *E. coli* ATCC 25922, *S. aureus* ATCC 6538, and *S. pyogenes* ATCC 19615, respectively.

Using a similar procedure, Haney et al. (2018) found that media composition can inhibit and even eradicate the biofilm formation of *P. aeruginosa* and *S. aureus* [44]. Using various procedures, previous papers did not study the antimicrobial and antibiofilm effects of natural Ɣ-PGA NP. Ghosh et al. (2021) found that conjugated polymer nanostructure polythiophene (PEDOT) could completely inhibit *S. aureus* and *E. coli* at 883 ng ml^−1^ and 333 ng ml^−1^, respectively [54]. Bactericidal activity was related to the nanofibrous structure of materials, as evidenced by inhibition of bacterial growth in exposure to bulk polymers. The light absorption and high surface area enhanced the biocidal activity of the NPs. Researchers studied doping nanoparticles or antibiotics inside a biodegradable polymer. Su et al. (2016) studied the antibiofilm effect of silver nanoparticles doped into carboxymethyl cellulose (CMC) and sodium alginate (SA) of sizes 30–60 nm against the antibiofilm of *K. pneumoniae* MTCC 4032 and *S. pyogenes* MTCC using the standard Kirby–Bauer disc diffusion method and agar well diffusion method. The antibiofilm efficacy of nanoparticle-doped polymers has high antibiofilm activity. They concluded that increasing their nanocomposites concentrations decreased biofilm formations [55]. Also, other studies focused on studying the antimicrobial activity of antibiotic-encapsulated polymers. Cheow et al. (2010) studied using PLGA and PCL as drug carriers for ciprofloxacin and levofloxacin antibiotics against *E. coli* biofilm. They found that MBIC was 0.90 and 0.15 μg/mL for ciprofloxacin and levofloxacin, respectively [56]. Antibiotic-loaded polymer nanoparticles have been detected for their high physical characteristics and antibiofilm efficacy, so they could be used as drug delivery vehicles in the treatment of bacterial biofilm with low doses. Tokam et al. (2023) found that thymol-loaded PLGA nanoparticles showed that MBEC and MBIC of *Salmonella typhi*, *S. typhimurium*, and *S. choleraesuis* were reduced by 32 to 128-fold [57], which was more effective than (8 to 32 times) free thymol. This is because of the high interaction between them and biofilms.

Hemmati et al. (2020) used Ch-ZnO of size 277.5 nm as an antibiofilm agent against *S. aureus* and *P. aeruginosa.* They found that Ch-ZnO reduced *P. aeruginosa* biofilm by 84% and the biofilm formation of *S. aureus* by 77% [58]. That is because of the synergistic action of Ch-ZnO nanocomposite, which led to the development of a new combined therapy against biofilms.

Others used nanocomposites, nanoparticles, or antibiotics as antibiofilm, such as Lu et al. (2018), who proved that the biofilm formation of *S. aureus* and *E. coli* was reduced to 20–25% after affecting Ch-Au@MMT nanocomposites (128 μM), and the density of CV staining decreased along with increasing concentrations of Ch-Au@MMT, indicating successful inhibition of biofilm formation [46]. The cationic amine allowed the nanocomposite to transport to the negative charges in the bacterial cells through electrostatic adhesion, with synergistic effects from the nanoparticles, and then observed a strong bactericidal inhibition for biofilms. Moreover, the nanocomposite can adhere to the biofilm rupture by rendering bacterial cells inactive. This facilitated Ch-Au@MMT penetration of the biofilm, leading to sustained damage and biofilm elimination. Also, Tassew et al. (2017) proved that many antibiotics failed to inhibit *Mycoplasma hyopneumoniae* biofilm at concentrations 10-fold higher than the MICs in planktonic cultures [59]. They observed that the antibiotics failed to inhibit the rough *Mycoplasma hyopneumoniae* polysaccharide production in the biofilm cells.

Some previous works studied the antibiofilm effect of some nanoparticles against clinical and reference strains. Shariati et al. (2019) used nano-curcumin as an antibiofilm against clinical strains of *P. aeruginosa* and its reference strain PAO1. They found that *P. aeruginosa* PAO1 biofilm was reduced more than the clinical strains at the same nano-curcumin concentration. That was because of the greater effect of nano-curcumin on decreasing the expression of the virulence gene and biofilm formation [52]. Also, Ansarifard et al. (2021) found that CuO nanoparticles were more effective against the biofilms of *Candida albicans* (CBS 10261, 1905, 1912, 1949, 2730), *S. mutans* (ATCC35668), *S. sobrinus* (ATCC27607), and *S. salivarius* (ATCC9222) than their clinical isolates [60]. From previous studies, it is clear that the structure of the cell wall plays an important role in the tolerance or susceptibility of the microorganism in the presence of metal NPs. Moreover, the biofilm structure of reference strains was more fragile than that of clinical strains. So, the nanoparticles can inhibit reference strains’ biofilms more than clinical strains’ biofilms at the same concentration.

Differences between Gram-positive and negative bacterial cell walls may cause the differences in antibiofilm response to NPs, as Gram-positive bacteria are surrounded by a thick peptidoglycan cell wall, while Gram-negative bacteria are surrounded by a thin peptidoglycan cell wall with an outer membrane containing lipopolysaccharide.

### 3.3. Assessment of Antibiofilm by Cell Counting

After biofilm cells were suspended by vigorous pipetting for 1 min, their CFU/μL was counted as shown in Table 2. The counted number was not the same in MBEC and MBIC values. Bacterial reduction using the MBEC technique showed a lower CFU number compared with that of MBIC at the same time. Our general observation was that the bacterial strains were viable in the wells until 8, 12, 12, and 24 h for *S. pyogenes*, *S. aureus*, *E. coli*, and *K*. *pneumoniae*, respectively. After that time, no bacterial growth was detected. This may contribute to the bacterial eradication after that time of exposure with the Ɣ-PGA NPs. The difference in time between bacterial strains for bacterial eradication may be due to the much more structured biofilm with complex shapes for *K*. *pneumoniae* than for *E. coli* and *S. aureus* than for *S. pyogenes.*

Moreover, the CFU of reference strains was lower than that of clinical strains. This was a result of the high efficiency of Ɣ-PGA NPs against the reference strains at the same concentration.

Also, Kragh et al. (2019) found that scraping and ultra-sonication removed the same number of cells as scraping alone. They also found that CFU of *P. aeruginosa* biofilm treated with tobramycin was reduced by approximately 50% from 24 to 48 h and by a further 50% from 48 to 72 h as the fraction of the biomass bound in aggregates in the bottom of the wells continued to grow in biomass over 72 h [61]. Moreover, they found that when ultra-sonication was used to remove biomass from the surface, there was still visible biomass at the edges of the wells [61].

Costa et al. (2017) noticed that Ch NPs reduced three log cycles of CFU *S. aureus* biofilm and a reduction of viable cells to levels below the method’s detection limit (2.69 log CFU/mL) within the first hour of the assays [62]. Ch NPs could be tightly adsorbed on the bacterial cell surfaces due to their large surface area, thus leading to well disruption around the bacterial cells. Ch NPs lower quantity of aggregates and higher population homogeneity let them be more effectively distributed and adsorbed around the bacterial cell.

Moreover, Cruz et al. (2018) showed that a 3–4 log10 CFU reduction was achieved by treating biofilm cells of *E. faecalis* ATCC 35667 and *E. faecalis* VRE ATCC 51575 with 160 μg/mL ciprofloxacin, respectively [8]. It was proven that the presence of media containing carbohydrates (such as TS media) plays an important role in the biofilm production of Gram-positive bacteria. The development of new antibiofilm agents lacks a reliable method for screening the antibiofilm activity (MBEC and MBIC).

In the same context, Santhoth et al. (2015) showed that epoxy/Ag-TiO_2_ with 1.0 wt% loading was found to cause a reduction of CFU on agar plates by approximately 6-log in the case of *E. coli* and a 4-log reduction in the case of *S. aureus* after 48 h of incubation, while epoxy/TiO_2_ with 1.0 wt% loading exhibited lesser inhibition of biofilm formation [63]. These antimicrobial agents could inhibit microorganisms by inhibiting biofilm formation in an aqueous environment.

### 3.4. Analysis of Biofilm Vitality and Thickness by CLSM

**Figure 2:**
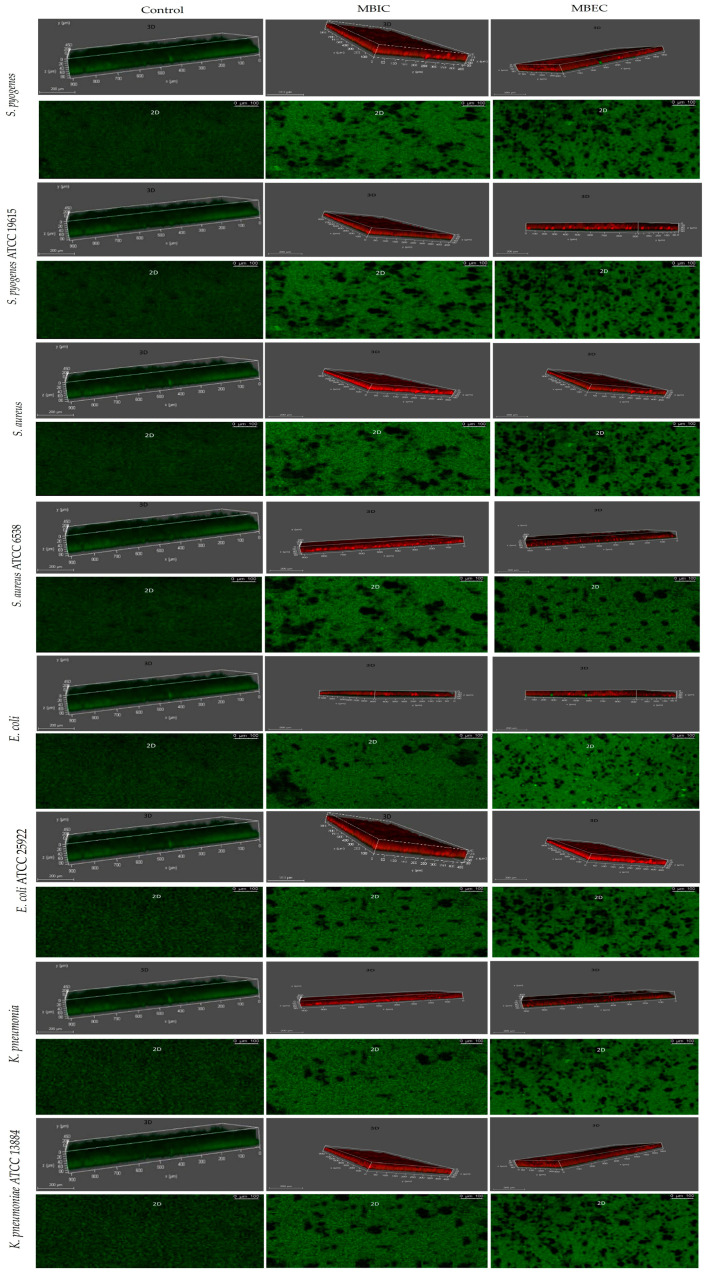
(CLSM) characterization of the biofilms of the stained strains of the clinical and reference strains.

The CLSM view showed strong biofilm formation in the bacterial strains, whereas Ɣ-PGA NPs reduced biofilm formation. A significant reduction in biofilm growth was observed after exposure for 24 h. On the other hand, the effect of Ɣ-PGA NPs against all the tested bacterial biofilms showed decreasing numbers in the living bacterial count. As shown in Figure 2, it is obvious that the Ɣ-PGA NPs effect was more against *S. pyogenes* than *S. aureus* than *E. coli* than *K. pneumoniae*, which justifies the results obtained by ELISA readings and CFU results. Also, living bacterial cells at MBEC decreased to 80%, while at MBIC they decreased up to 60% with all clinical bacterial strains. These data justify those of CFU quantification for *E. coli* and *K. pneumoniae*. On the other hand, CFU and CV quantification showed that bacterial viability in the wells was inhibited and eradicated after 24 h for *S. pyogenes* and *S. aureus*, and their CLSM results show their viability after 24 h. This heterogeneous growth may contribute to the deviations often found in this model, as the local microenvironment in each well may influence growth and strength.

Also, CLSM photos of the reference strains showed strong biofilm formation in the bacterial strains, whereas Ɣ-PGA NPs reduced biofilm formation. The reduction in biofilm growth was observed after exposure for 24 h. Also, the effect of Ɣ-PGA NPs against all the tested bacterial biofilms showed decreasing numbers in the living bacterial count. As shown in Figure 2, it is obvious that Ɣ-PGA NPs were more effective against *S. pyogenes* ATCC 19615 than *S. aureus* ATCC 6538, *E. coli* ATCC 25922, and *K. pneumoniae* ATCC 13884, which justify the results obtained by ELISA readings and CFU results. Moreover, living bacterial cells at MBEC decreased to 70%, while at MBIC, they decreased up to 50% with all bacterial reference strains. These data justify those for CFU quantification. On the other hand, CFU and CV quantification showed that bacterial viability in the wells was inhibited and eradicated after 24 h for the four reference strains, and their CLSM results showed their viability after 24 h. So, the live cells were counted using ImageJ software, which was more accurate than CFU, as shown below.

As mentioned by Kragh et al. (2019), scraping and ultra-sonication have been recommended for removing attached biomass from microtiter wells [61].

Using CLSM, Ali et al. (2019) tested Au nanoparticles against *P. aeruginosa* and found that the biofilm formation was inhibited up to 59.9% at 150 μg/mL, 36.6% at 100 μg/mL, and 27.1% at 50 μg/mL [64]. Increasing Au nanoparticle concentrations decreases the number of *P. aeruginosa* biofilm-forming cells. Furthermore, the interaction between Au nanoparticles and *P. aeruginosa* increases the cell’s ability to adsorb nanoparticles on its surface.

Vijayakumar et al. (2019) noticed that using 100 μg mL^−1^ of Ch-Ag nanoparticles caused a reduction in biofilm formation by 40% and 70% of *P. aeruginosa* and MRSA, respectively [65]. Ch-Ag nanoparticles have high biofilm detachment activity against Gram-negative bacteria compared with Gram-positive ones. Also, the biofilm formed in the presence of Ch-Ag nanoparticles detached more easily with surfactant than those without Ch-Ag nanoparticles. That is because Ch-Ag nanoparticles have excellent biocompatibility with microbial cells.

Also, Ansari et al. (2013) proved that the biofilm formed by *E. coli* is reduced approximately by 20% after the first day of incubation with Ag nanoparticles [66]. The complete antibiofilm activity of Ag nanoparticles was at concentrations up to 50 μg/mL. The nanoscale level of the silver particles increases the surface area, which leads to a higher Ag+ rate release than for elemental Ag+ particles. Also, nanoparticles have a high capacity to attach, penetrate, and accumulate inside bacterial cells, providing a release of Ag+ inside the bacterial cell.

Using clinical isolates of *P. aeruginosa*, Ali et al. (2018) used Ag nanoparticles as antibiofilm agents; they also imaged the effect using CLSM after incubation for 24 h. They concluded through the CLSM analysis that as the concentration of the nanoparticles increases, the biofilm cells decrease, and the cells no longer remain adhered to the surface due to the reduction in the survival of cells [67]. Ansari et al. (2015) used CLSM to study the Ag nanoparticles effect against biofilms of some clinical isolates of *S. aureus* and *S. epidermidis*. They observed a complete absence of clumped cells in the presence of 25 μg/mL Ag nanoparticles [68]. On the other hand, using CLSM, Nair et al. (2018) used biofilms of reference strains of *Enterococcus faecalis* (ATCC 29212, OG1RF) to investigate the effect of ZnO nanoparticles and Ch NPs. They proved that the biofilm thickness was reduced, their architecture was changed, and the viable bacterial cells were reduced [69].

**Figure 3:**
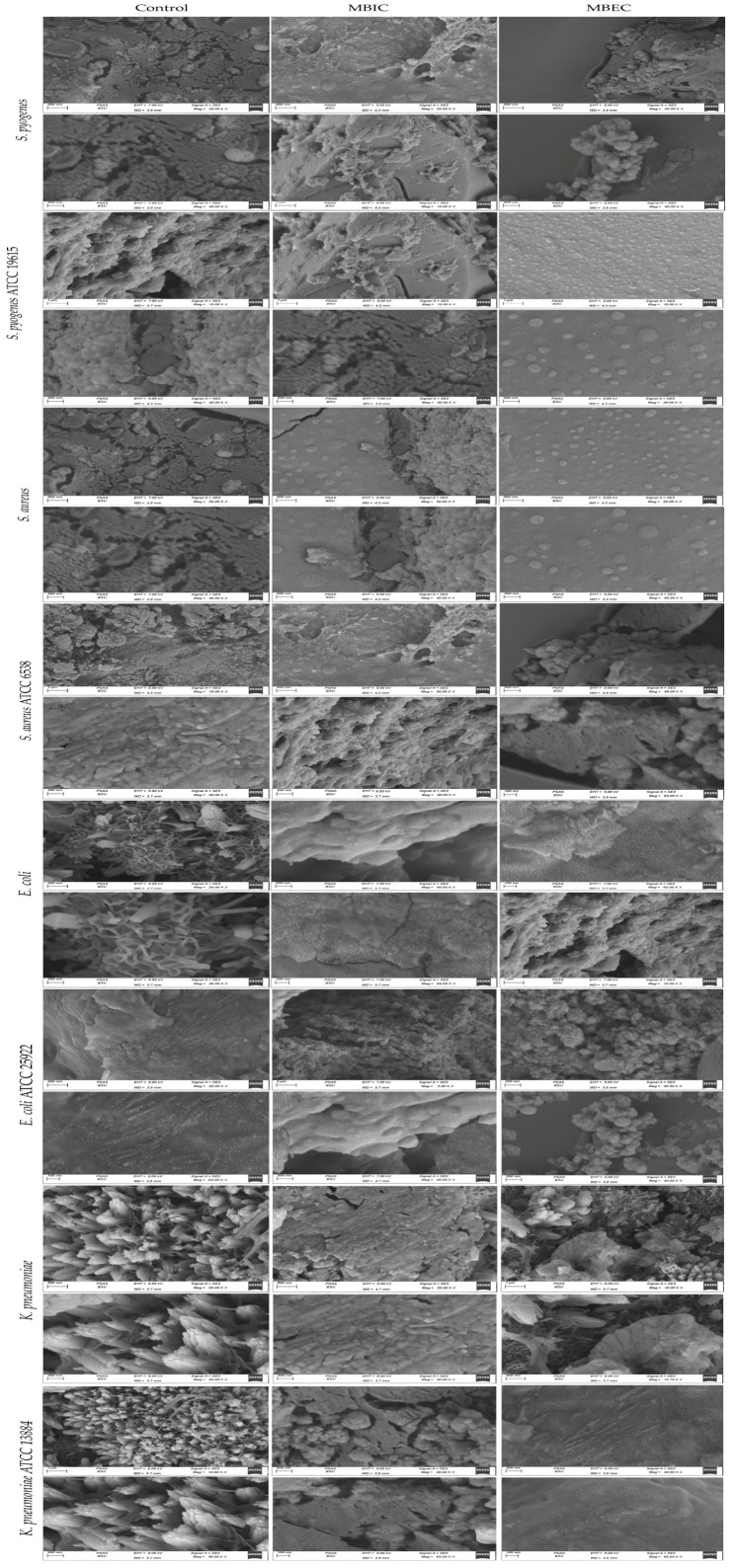
(SEM) characterization of the biofilms of the clinical and reference strains before and after Ɣ-PGA NPs exposure.

As observed in Figure 3, significant removal of bacterial biofilms was seen after treatment with Ɣ-PGA NPs, and fewer clusters of individual cells remained attached compared with the control. Moreover, SEM images prove that the Ɣ-PGA NPs effect was more against *S. pyogenes* than *S. aureus* than *E. coli* than *K. pneumoniae*. Also, Ɣ-PGA NPs were more effective at MBIC than MBEC, i.e., bacterial colonization is lower at SEM images for MBIC than MBEC.

As discussed by Ma et al. (2020), who proved that curcumin (Cur) loaded on positively charged Ch nanoparticles had a great effect against *Candida albicans*, these nanoparticles reduced polymicrobial biofilms, which displayed more free regions without adhered cells [70].

Biofilms grown without NPs showed normal cellular morphology with smooth cell surfaces. Also, microbial cells were arranged in an exopolysaccharide matrix. Ɣ-PGA NPs penetrated the bacterial biofilm and damaged the microbial cells. As described by Dorgham et al. (2022), the presence of Ag nanoparticles, the cell morphology change of *S. aureus* ATCC 6538 and *C. albicans* DAY185 biofilms, and the bacterial colonization on the surfaces were inhibited [71]. In the same context, the small size of Ɣ-PGA NPs increases their ability to attach and penetrate bacterial biofilms, which then enhances the antibiofilm activity, which leads to the removal of the biofilm in MBIC fields during SEM examination. This agrees with El-Telbany et al. (2022), who affected *P. aeruginosa* using ZnO nanoparticles and revealed complete inhibition of treated biofilms [72].

Also, as observed in Figure 3 with reference strains, bacterial biofilms were significantly removed after treatment with Ɣ-PGA NPs, and fewer clusters of individual cells remained attached compared with the control. SEM images prove that the Ɣ-PGA NPs effect was more against *S. pyogenes* ATCC 19615 than *S. aureus* ATCC 6538 than *E. coli* ATCC 25922 than *K. pneumoniae* ATCC 13884. Moreover, Ɣ-PGA NPs were more effective at MBIC than MBEC. It is obvious that SEM images show the greater efficiency of Ɣ-PGA NPs against reference strains than clinical strains, as reported in many previous experiments.

As discussed by Raouf et al. (2021), *P. aeruginosa* isolates and the reference strain PAO1 ATCC 27853 biofilms were affected by ciprofloxacin-azithromycin nanoparticles on the Ch nanocarrier (Cipro-AZM-Ch). SEM images showed that the sample treated with Cipro-AZM-Ch had the least number of biofilm-forming cells [73]. Also, Miglani and Tani-Ishii (2021) affected *Enterococcus faecalis* (MTCC 439) biofilm using selenium nanoparticles. SEM images proved the greatly inhibited biofilm [74].

### 3.5. ImageJ Counting

**Figure 4:**
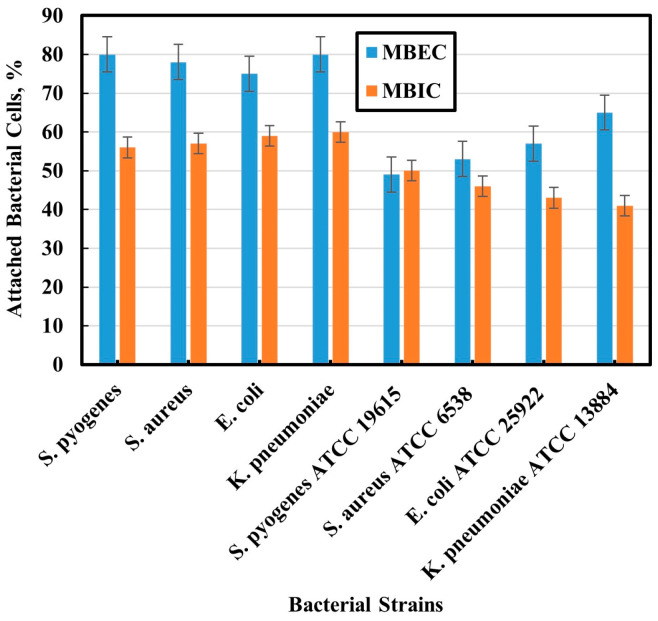
Percent of 2D bacterial cells using ImageJ software.

Through quantitative observation of the stained bacteria using ImageJ software, control images were compared with MBIC and MBEC images. As shown in Figure 4, MBIC and MBEC decreasing bacterial percent agree with that concluded by CLSM images. There is a difference between manual (CFU) and software counting of live cells. The same results were obtained for the MBIC and MBEC of the reference strains. Their MBIC and MBEC decreased, which agrees with the results concluded by CLSM images. Also, there is a difference between the manual (CFU) and the software counting of live cells. As shown in Figure 4, cell counting was also less than that of clinical strains because of the greater efficiency of the NPs against the reference bacterial biofilms.

Some prior studies have directly compared the image analysis of different simple software with traditional quantification methods. Singh et al. (2011) and Mountcastle et al. (2021) used ImageJ software for counting live cells [45,75]. They also confirmed the variation in live cell count between the manual counting and image analysis methods. Using manual techniques such as CFU counting results in larger errors. This may be due to the increased number of cells present in the larger biofilms, making counting manually less accurate.

### 3.6. In Vivo Anti-Biofilm Efficiency of Ɣ-PGA NPs

**Figure 5:**
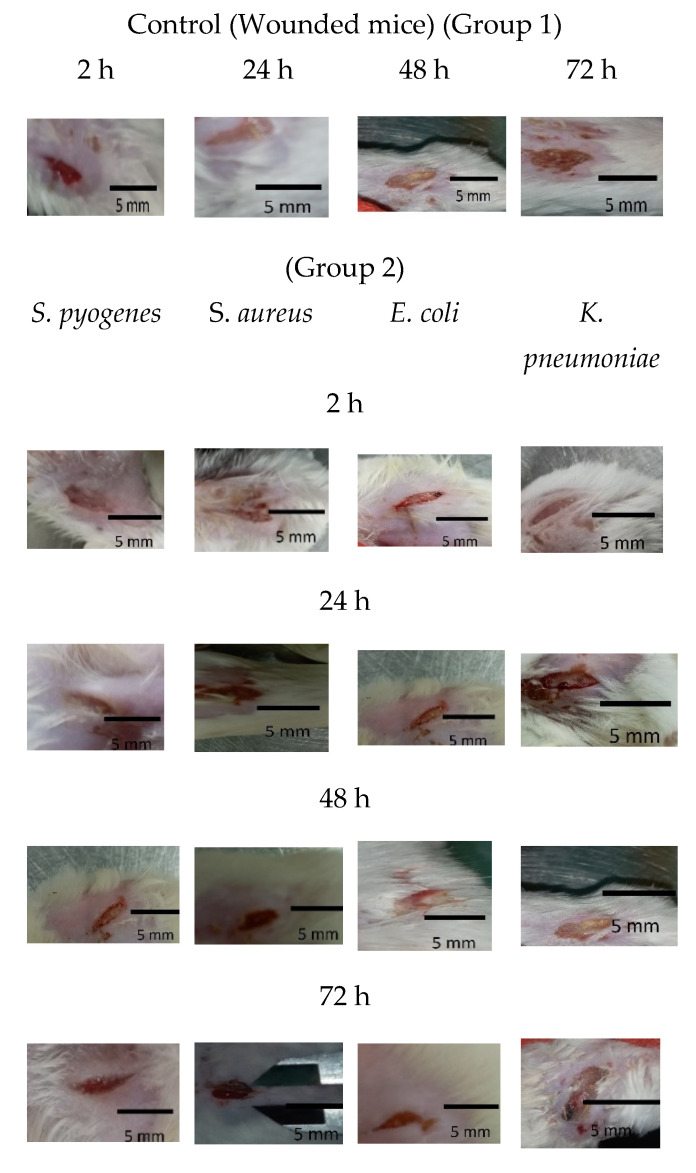

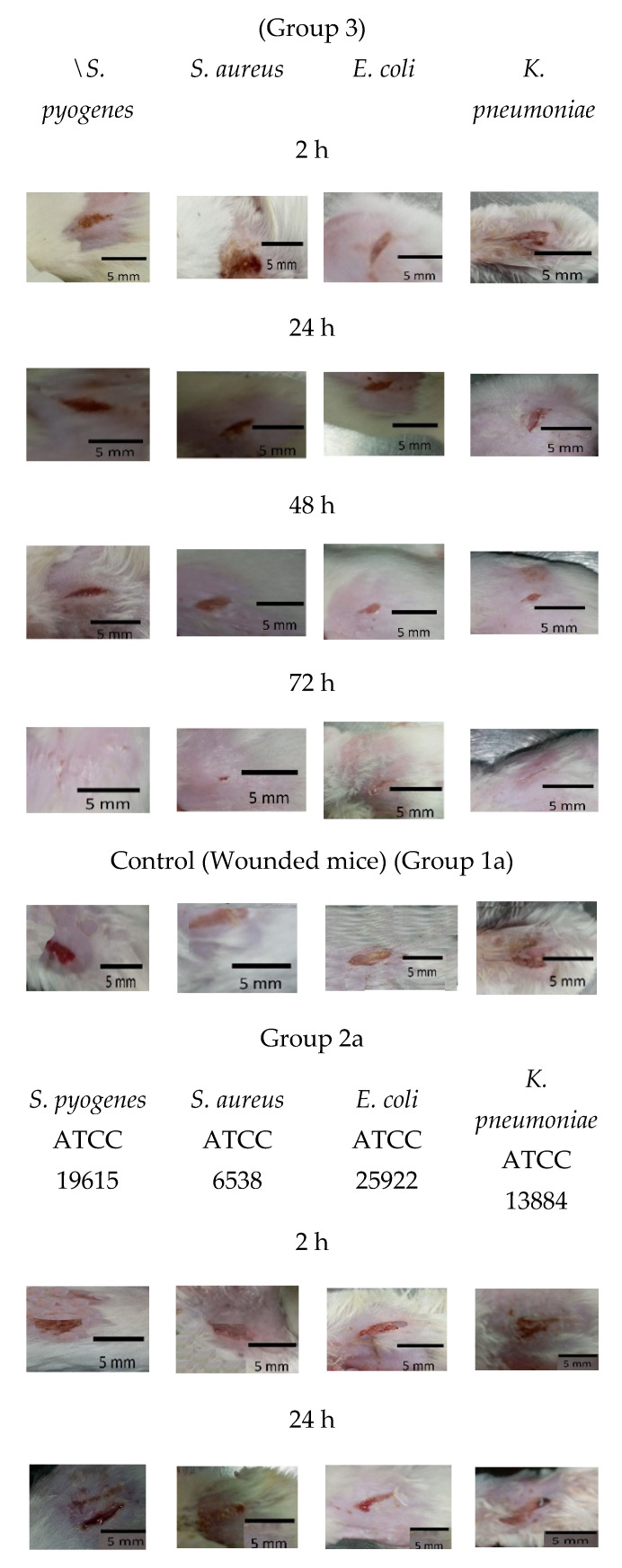

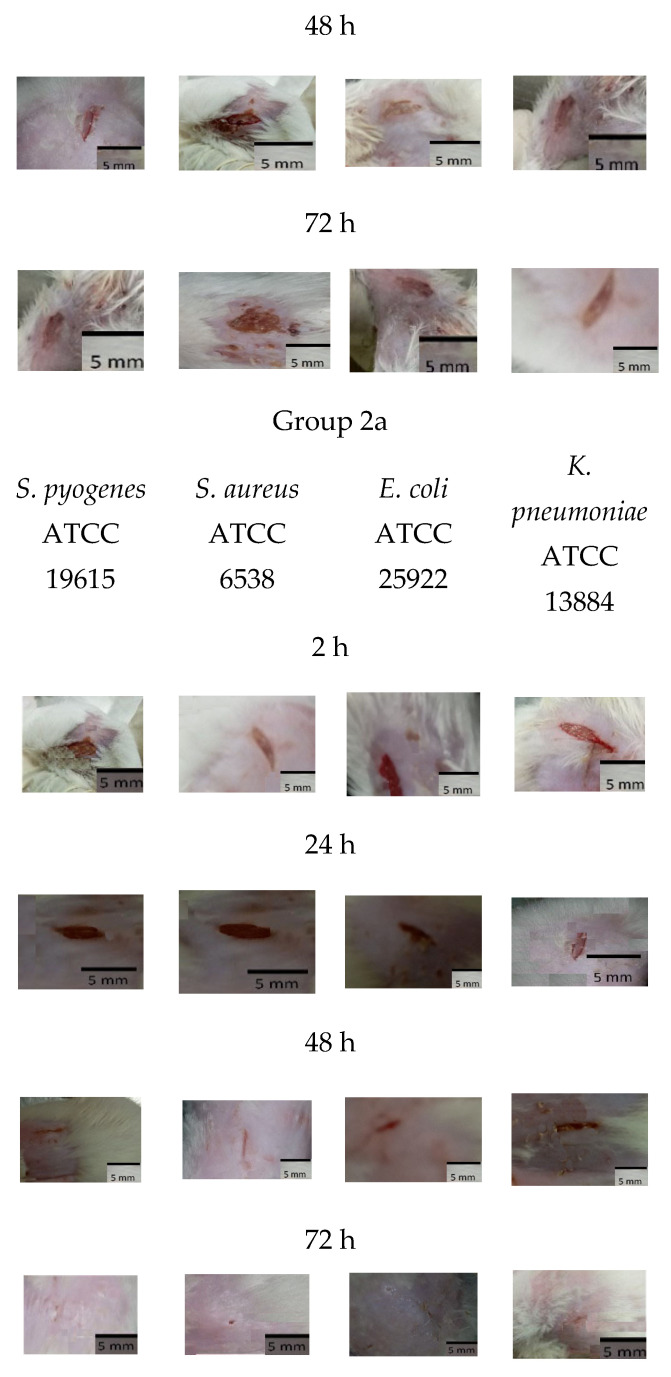
Group 1 mice, 2 mice, 3 mice, 1a mice, 2a mice, and 3a mice.

**Figure 6:**
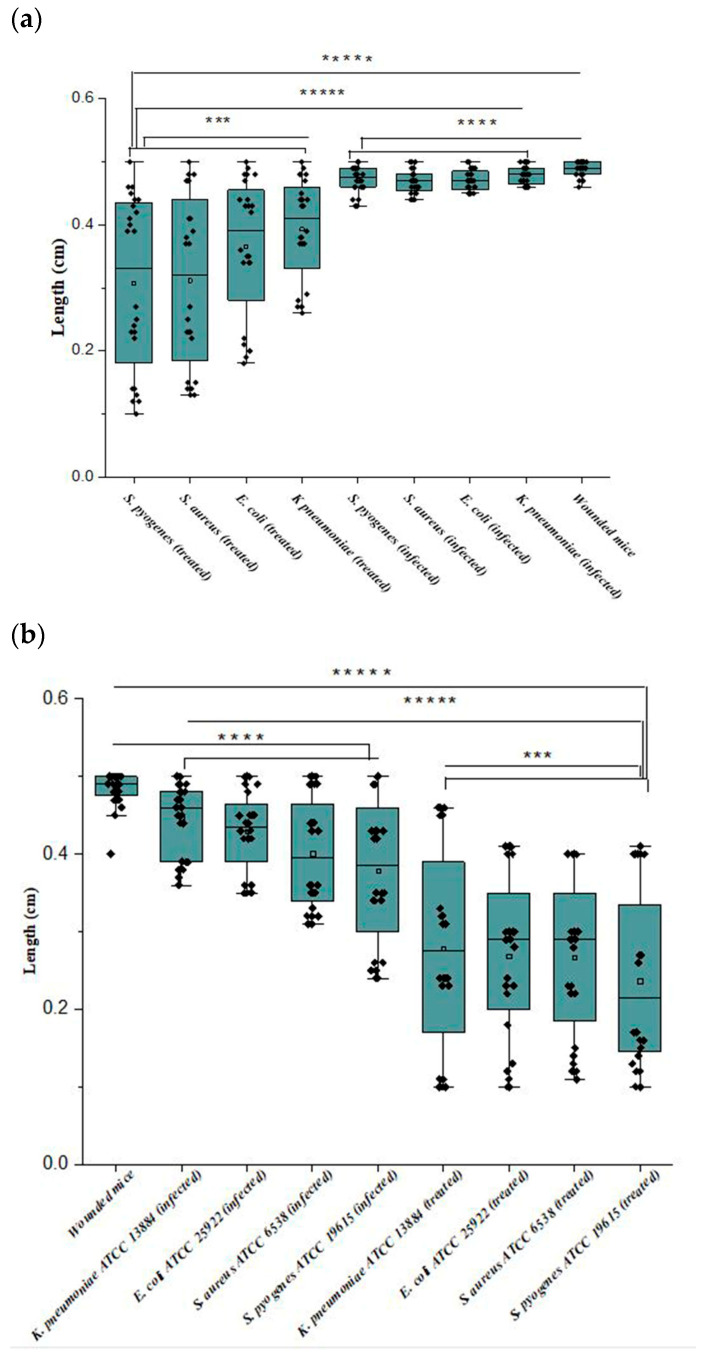
(**a**,**b**) Wound lengths of the mice infected with clinical bacterial strains and mice infected with reference strains; where ***** *p* < 0.00001; **** *p* < 0.0001; *** *p* < 0.001.

As shown in Figure 5 and Figure 6a, mice treated with Ɣ-PGA NPs (group 3) showed faster treatment and wound healing (*p* < 0.00001) than those that were only infected without treatment (group 2), and those (*p* < 0.00001) that were only wounded (group 1). Also, wounds in group 1 mice were treated faster (*p* < 0.0001) than in group 2.

Briefly, the infected mice in group 3 were treated faster (*p* < 0.0001) when infected by *S. pyogenes* > *S. aureus* > *E. coli* > *K. pneumoniae*. Wound lengths after 2 h showed minute treatment (*p* < 0.001), while treatment became more obvious after 72 h > 48 h > 24 h (*p* < 0.0001), as wounds began to heal after 24 h up to 72 h.

At group 2, wounds were still infected with the bacterial biofilm till 72 h, and the infection increased in all the mice up to 48 h. Also, mice infected by *K. pneumoniae* showed the slowest healed wounds, followed by *E. coli*, S. *aureus*, and *S. pyogenes*. That trend was the same as the treatment in group 3 mice.

Wound treatment using Ɣ-PGA NPs in group 3 allowed wound healing to be more efficient than in group 2 mice.

For reference strains, as shown in Figure 5 and Figure 6b, mice treated with Ɣ-PGA NPs (group 3a) showed faster treatment and wound healing (*p* < 0.00001) than mice that were only infected without treatment (group 2a) and those (*p* < 0.00001) that were only wounded (group 1a). Moreover, wounds in group 1a mice were treated faster (*p* < 0.0001) than in group 2a.

Briefly, the infected mice in group 3a were treated faster (*p* < 0.0001) when infected by *S. pyogenes* ATCC 19615 > *S. aureus* ATCC 6538 > *E. coli* ATCC 25922 > *K. pneumoniae* ATCC 13884. After 2 h, wound lengths showed minute treatment (*p* < 0.001), while treatment became more obvious after 72 h > 48 h > 24 h (*p* < 0.0001), as wounds began to heal after 24 h up to 72 h.

For group 2a, wounds were still infected with the bacterial biofilm till 72 h, the same as mice infected with the clinical bacterial biofilm, and the infection increased in all the mice up to 48 h. Also, mice infected with *K. pneumoniae* ATCC 13884 showed the slowest healed wounds, then *E. coli* ATCC 25922, then *S. aureus* ATCC 6538, and then *S. pyogenes* ATCC 19615. That trend was the same as the treatment in group 3a mice.

Although group 1 also showed slowly decreased wounds, pus, ulceration, and edema could still be observed on the wound sites up to 72 h, demonstrating poor inhibition of wound biofilm.

After 72 h, group 2 wound puss almost began to decrease except for *S. aureus*, which began to decrease after 48 h, while group 2a wound puss began to decrease after 24 h. At 24 h post-surgery, group 3 wound puss began to decrease from biofilm infection, indicating the high efficiency of Ɣ-PGA NPs to eradicate the wound bacterial biofilm. Notably, mice infected with *K. pneumoniae* in this group began to be treated after 48 h. For group 3a mice, at 2 h post-surgery, their wound puss began to decrease from the biofilm infection for all treated mice.

**Figure 7:**
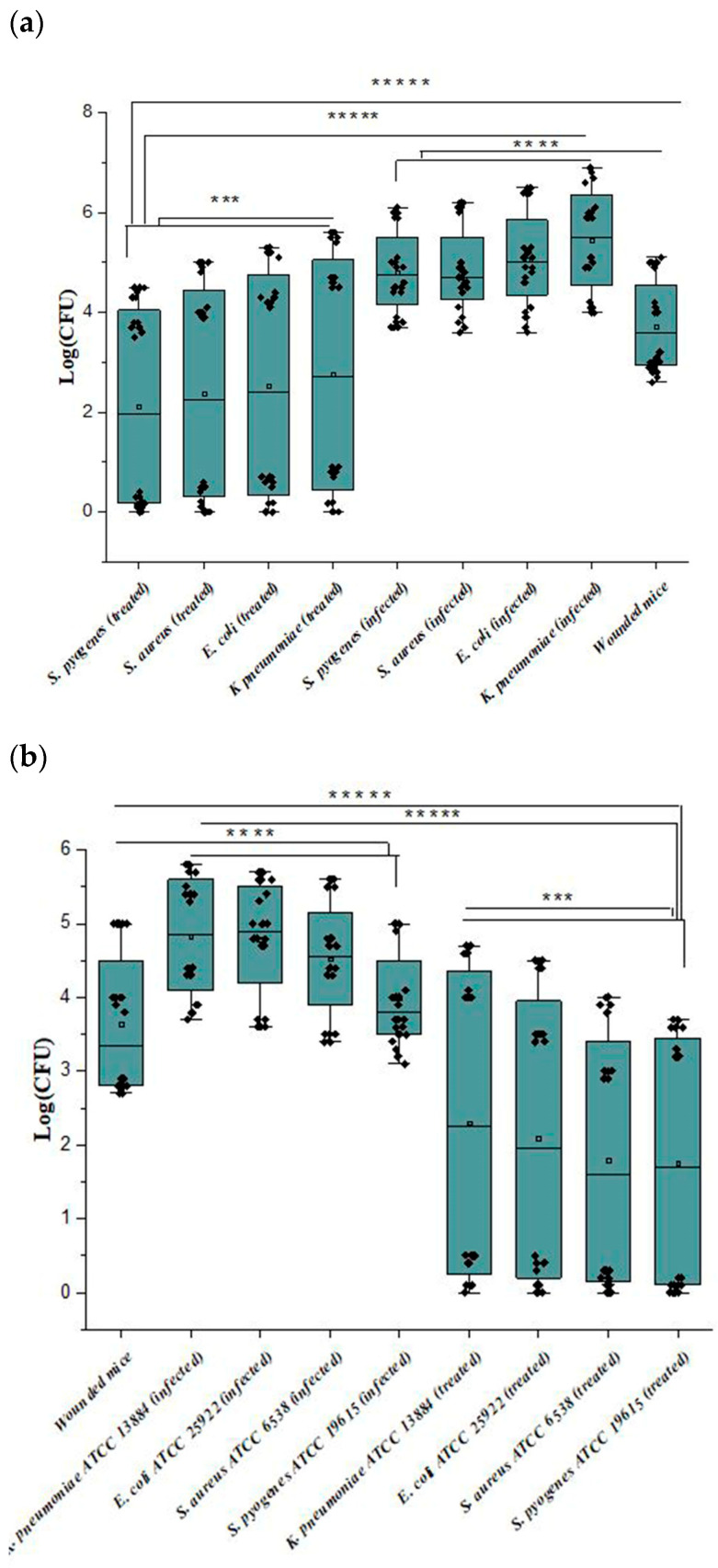
(**a**,**b**) Wound bacterial count of the mice infected with the clinical bacterial strains and mice infected with the reference strains, where ***** *p* < 0.00001; **** *p* < 0.0001; *** *p* < 0.001.

As shown in Figure 7a, bacterial colonies (CFU) detached from the wounds revealed that the bacterial count was more abundant in group 2 > group 1 > group 3. Also, CFU detached from the wounds revealed that the bacterial count was more abundant in group 2a > group 1a > group 3a, as shown in Figure 7b. The bacterial count decreased in groups 3 and 3a with increasing time of treatment, while the bacterial count in group 2 was not affected up to 72 h. Also, CFU for group 2a was affected after 48 h. Group 1 and group 1a bacterial counts were low as wounds were contaminated because they were not treated with any treatment.

Similarly, the bacterial count of mice infected with *S. aureus* in group 2 showed a relative decrease after 48 h compared with other infected mice at that time. Also, the CFU of mice infected with *K. pneumoniae* in group 3 did not significantly decrease after 24 h. So, wounds’ length decreased to 1, 1.1, 0.9, and 0.8 mm for *K. pneumoniae*, *E. coli*, *S. aureus*, and *S. pyogenes*, respectively.

For group 2a, the CFUs of all four bacterial biofilm wounds showed a relative decrease after the same time (48 h). Group 3a wound length decreased up to almost 1 mm for all treated mice.

It is important to point out that group 2a showed more rapid healing than group 2, and group 3a showed faster healing than group 3a.

These results supported the in vitro results, where Ɣ-PGA NPs showed superior antibacterial and anti-biofilm efficiencies.

Some papers focused on applying nanocomposites, drug carriers’ nanoparticles, or one type of nanoparticle as antibiofilm in vivo. Choi et al. (2020) in vivo applied Ch and Ch-based nitric acid (Ch/NO)-releasing dressing as antibiofilm against MRSA. They showed that the bacterial viability in the untreated mice group increased from day 6 to 15 post-injury. The Ch film and Ch/NO film-treated groups showed that the biofilm in the Ch film-treated group tended to increase, whereas in the Ch/NO film-treated group it started to decrease. So, Ch/NO films enhanced wound healing in MRSA biofilm-infected wounds [14]. The Ch/NO could disperse the biofilm, reduce wound size, and deposit collagen faster than the only Ch-affected wounds and the untreated groups. Therefore, Ch could be effectively used as a carrier for NO to treat wounds faster.

Kulshresth et al. (2016) found a significant reduction of *S. mutans*, and hence a reduction of caries, in rats treated with CaF_2_-NPs as compared with the control [4]. These nanoparticles could affect the acid production and the exopolysaccharides of the bacterial biofilm; hence, they could reduce the caries of *S. mutans*. Also, Lu et al. (2018) results showed that rabbits injured and infected with *S. aureus* formed biofilm and began to form crust after 14 days of treatment with Ch-Au@MMT. Also, they proved that Ch-Au@MMT showed complete recovery from biofilm infection after 14 days [46]. The cationic amine of Ch-Au@MMT allowed its transport to the negatively charged surfaces of bacterial biofilms through electrostatic adsorption, allowing the synergistic effects of Au nanoparticles and Ch that exert a strong bactericidal effect against the biofilm. Also, mature biofilms could attract Ch-Au@MMT to their surfaces to inactivate bacterial cells, resulting in biofilm rupture. This allowed Ch-Au@MMT to penetrate through the biofilm, leading to continuous damage and biofilm elimination.

Raghuwanshi et al. (2017) used a nano-gold formulation (10–12 nm) to prevent microbial biofilm in order to accelerate wound healing in Wistar albino rats with 2 cm diameter wounds. The experiment showed the initiation of healing on the 3rd day of treatment with a diameter of 14.24 ± 0.26 mm^2^ [76]. Au nanoparticles are effective in preventing microbial adhesion, leading to sustained wound healing. Treatment of wounds and wounded tissue using Au nanoparticles results in quick aggregation of granular tissue, collagen fibrils, and repairing epithelial tissue, leading to rapid healing and closuring of wounds.

Some previous studies focused on using only reference strains. In one of those studies, Chari et al. (2017) examined Cu nanoparticles (100 ng/mL) against *Vibrio alginolyticus* (ATCC 17749), *Vibrio parahaemolyticus* (ATCC 17802), and *Aeromonas hydrophila* (ATCC 7966) for 24 h in Nauplii of *Artemia salina.* They found that the survival rate in the animals gives evidence that Cu nanoparticles are not toxic to the tested animal model and affect bacterial infection [77]. Also, Muthamil et al. (2018) studied the in vivo effect of Zn nanoparticles against biofilm infection of *Chromobacterium violaceum* ATCC 12472, *E. coli* ATCC 25922, *P. aeruginosa* PAO1, *K. pneumoniae* ATCC 700603, *Serratia marcescens* ATCC 13880, and *Listeria monocytogenes* (laboratory strain). They concluded that Zn nanoparticles combat drug-resistant bacterial infections and prevent contamination/spoilage of food [78]. Cheng et al. (2013) studied the in vivo effect of the releasing Ag from Ag-incorporated nanotubes against *S. aureus* (MRSA, ATCC43300), which showed a very good antibacterial activity over 30 days [79].

It is concluded from the in vitro and in vivo tests of our study that clinical strains are more resistant than reference strains. This is because the most resistant germs are more common in hospitals than in the community. Also, the overuse of antibiotics in recent years has led to the appearance of microorganisms that are more resistant to antimicrobial agents. Besides, organic materials affect the antimicrobial activity of the nanoparticles in vivo. Moreover, microorganisms change their gene expression to activate resistance mechanisms that protect them from antimicrobial threats, especially in the case of biofilms.

## 4. Conclusions

In this study, Ɣ-PGA NPs were used as a biofilm agent. They showed good activity against Gram-positive and negative clinical and reference strains. Furthermore, the inhibitory effect of Ɣ-PGA NPs showed good inhibition using the MIC technique. Moreover, the in vitro antibiofilm activity of Ɣ-PGA NPs at both MBEC and MBIC increased with increasing their concentration or time of exposure. Notably, the MBIC values of Ɣ-PGA NPs were lower than those of MBEC. Moreover, MBEC values showed that Ɣ-PGA NPs concentrations affected *K. pneumoniae* at higher concentrations than *E. coli*, *S. aureus*, and *S. pyogenes.* Of note, MIC, MBEC, and MBIC for the reference strains were lower than those of the clinical strains. Also, MBEC values showed that *K. pneumoniae* ATCC 13884 was more resistant to Ɣ-PGA NPs than *E. coli* ATCC 25922, *S. aureus* ATCC 6538, and *S. pyogenes* ATCC 19615. Those results were confirmed using CLSM and SEM. In vivo experiments showed the well-healing effect of Ɣ-PGA NPs. Also, treatment was observable with increasing time when mice were infected with *S. pyogenes*, *S. aureus*, *E. coli*, and *K. pneumoniae*. Briefly, the infected mice were treated faster when infected by *S. pyogenes* ATCC 19615 > *S. aureus* ATCC 6538 > *E. coli* ATCC 25922 > *K. pneumoniae* ATCC 13884. Also, group 2a showed a faster healing than group 2, and group 3a showed a faster healing than group 3a.

## Figures and Tables

**Table 1:** MIC determined by Ɣ-PGA NPs. The result was represented as mean of the sample concentrations (μg/mL) with zero SD. Number of detected samples in each group is three.

Bacterial Strain	MIC (μg/mL)	
	Ɣ-PGA NPs	Standard (Gentamicin)
*K. pneumoniae*	3.2	12.8
*E. coli*	1.6	0.8
*S. aureus*	0.4	0.4
*S. pyogenes*	0.2	0.2
*K. pneumoniae* ATCC 13884	1.6	3.2
*E. coli* ATCC 25922	0.8	1.6
*S. aureus* ATCC 6538	0.2	0.4
*S. pyogenes* ATCC 19615	0.2	0.4

**Table 2:** MBIC and MBEC determined by Ɣ-PGA NPs and cell counting methods. Data are expressed as mean ± SD. Number of samples in each group is three.

Strain	Time (h)	MBEC (μg/mL)	CFU/μL	MBIC (μg/mL)	CFU/μL
*K. pneumoniae*	2	51.2 ± 0.02	220 ± 0.09	12.8 ± 0.01	160 ± 0.005
	4	51.2 ± 0.10	130 ± 0.07	6.4 ± 0.02	110 ± 0.006
	6	12.8 ± 0.01	110 ± 0.06	1.6 ± 0.022	80 ± 0.02
	8	6.4 ± 0.20	20 ± 0.03	0.8 ± 0.002	30 ± 0.008
	12	3.2 ± 0.025	2 ± 0.02	0.4 ± 0.01	2 ± 0.02
	24	1.6 ± 0.012	2 ± 0.02	0.4 ± 0.01	2 ± 0.10
	48	1.6 ± 0.022	Nil	0.2 ± 0.02	Nil
*K. pneumoniae* ATCC 13884	2	25.6 ± 0.10	200 ± 0.02	12.8 ± 0.10	130 ± 0.01
	4	12.8 ± 0.01	190 ± 0.24	6.4 ± 0.03	110 ± 0.02
	6	12.8 ± 0.02	120 ± 0.24	0.8 ± 0.02	80 ± 0.01
	8	3.2 ± 0.20	10 ± 0.02	0.8 ± 0.10	2 ± 0.01
	12	3.2 ± 0.20	2 ± 0.01	0.4 ± 0.04	Nil
	24	1.6 ± 0.30	Nil	0.2 ± 0.10	Nil
	48	0.8 ± 0.01	Nil	0.2 ± 0.025	Nil
*E. coli*	2	51.2 ± 0.015	90 ± 0.07	12.8 ± 0.014	150 ± 0.08
	4	51.2 ± 0.01	30 ± 0.06	3.2 ± 0.013	100 ± 0.07
	6	12.8 ± 0.02	3 ± 0.03	1.6 ± 0.021	50 ± 0.009
	8	6.4 ± 0.20	2 ± 0.03	0.8 ± 0.02	3 ± 0.11
	12	1.6 ± 0.21	2 ± 0.01	0.4 ± 0.22	2 ± 0.11
	24	0.8 ± 0.016	Nil	0.2 ± 0.21	Nil
	48	0.8 ± 0.021	Nil	0.2 ± 0.16	Nil
*E. coli* ATCC 25922	2	25.6 ± 0.12	80 ± 0.001	12.8 ± 0.015	130 ± 0.013
	4	12.8 ± 0.01	30 ± 0.009	6.4 ± 0.3	110 ± 0.06
	6	3.2 ± 0.01	10 ± 0.007	0.8 ± 0.03	30 ± 0.002
	8	1.6 ± 0.03	2 ± 0.005	0.4 ± 0.02	2 ± 0.0014
	12	1.6 ± 0.02	2 ± 0.21	0.2 ± 0.01	Nil
	24	0.8 ± 0.10	Nil	0.2 ± 0.10	Nil
	48	0.8 ± 0.02	Nil	0.1 ± 0.016	Nil
*S. aureus*	2	51.2 ± 0.15	110 ± 0.04	6.4 ± 0.01	130 ± 0.19
	4	51.2 ± 0.13	60 ± 0.04	3.2 ± 0.02	50 ± 0.10
	6	6.4 ± 0.10	20 ± 0.10	0.8 ± 0.02	3 ± 0.14
	8	3.2 ± 0.01	3 ± 0.01	0.4 ± 0.24	2 ± 0.11
	12	1.6 ± 0.02	2 ± 0.03	0.4 ± 0.02	Nil
	24	0.8 ± 0.24	Nil	0.2 ± 0.01	Nil
	48	0.4 ± 0.02	Nil	0.2 ± 0.11	Nil
*S. aureus* ATCC 6538	2	12.8 ± 0.02	90 ± 0.003	6.4 ± 0.02	90 ± 0.10
	4	12.8 ± 0.01	60 ± 0.002	3.2 ± 0.013	50 ± 0.20
	6	3.2 ± 0.03	10 ± 0.001	0.4 ± 0.013	2 ± 0.21
	8	3.2 ± 0.02	8 ± 0.11	0.2 ± 0.02	2 ± 0.03
	12	1.6 ± 0.02	3 ± 0.01	0.2 ± 0.01	Nil
	24	0.4 ± 0.03	Nil	0.2 ± 0.03	Nil
	48	0.4 ± 0.02	Nil	0.1 ± 0.12	Nil
*S. pyogenes*	2	51.2 ± 0.12	50 ± 0.06	6.4 ± 0.01	60 ± 0.12
	4	25.6 ± 0.02	30 ± 0.05	1.6 ± 0.01	10 ± 0.01
	6	6.4 ± 0.015	3 ± 0.01	0.8 ± 0.02	2 ± 0.11
	8	3.2 ± 0.014	2 ± 19	0.4 ± 0.11	Nil
	12	1.6 ± 0.02	Nil	0.2 ± 0.12	Nil
	24	0.4 ± 0.20	Nil	0.2 ± 0.11	Nil
	48	0.2 ± 0.24	Nil	0.1 ± 0.002	Nil
*S. pyogenes* ATCC 19615	2	6.4 ± 0.24	50 ± 0.01	3.2 ± 0.0012	50 ± 0.002
	4	6.4 ± 0.20	40 ± 0.12	1.6 ± 0.11	30 ± 0.12
	6	3.2 ± 0.2	30 ± 0.001	0.8 ± 0.2	10 ± 0.11
	8	3.2 ± 0.21	2 ± 0.21	0.4 ± 0.24	2 ± 0.0013
	12	0.8 ± 0.002	2 ± 0.11	0.2 ± 0.03	Nil
	24	0.4 ± 0.24	Nil	0.1 ± 0.2	Nil
	48	0.2 ± 0.22	Nil	0.1 ± 0.22	Nil

## Data Availability

This article includes all of the data generated or analyzed during this investigation.
